# Deep learning-guided attenuation and scatter correction of ^99m^Tc-MAA SPECT images: towards quantitative analysis in ^90^Y-SIRT

**DOI:** 10.1007/s12149-025-02152-2

**Published:** 2026-01-05

**Authors:** Zahra Mansouri, Yazdan Salimi, Nicola Bianchetto Wolf, Ghasem Hajianfar, Ismini Mainta, Valentina Garibotto, Habib Zaidi

**Affiliations:** 1 Division of Nuclear Medicine and Molecular Imaging, Geneva University Hospital, Geneva, CH-1211 Switzerland; 2 Centre for Biomedical Imaging (CIBM), Geneva, Switzerland; 3 Laboratory of Neuroimaging and Innovative Molecular Tracers (Nimtlab), Geneva University Neurocenter and Faculty of Medicine, University of Geneva, Geneva, Switzerland; 4 Department of Nuclear Medicine and Molecular Imaging, University of Groningen, University Medical Center Groningen, Groningen, Netherlands; 5 Department of Nuclear Medicine, University of Southern Denmark, Odense, Denmark; 6 University Research and Innovation Center, Óbuda University, Budapest, Hungary

**Keywords:** ^90^Y-SIRT, Radioembolization, SPECT/CT, Attenuation correction, Scatter compensation, ^99m^Tc-MAA, Deep learning

## Abstract

**Purpose:**

This study aimed to develop deep learning (DL) models for CT-free attenuation correction and Monte Carlo-based scatter correction in ^99m^Tc-macroagregated albumin (^99m^Tc-MAA) SPECT imaging, with the goal of enhancing quantitative accuracy for improved treatment planning and pre-therapy dosimetry in ^90^Y-selctive internal radiation therapy (SIRT).

**Materials and methods:**

Data from 222 patients who underwent ^99m^Tc-MAA SPECT imaging prior to ^90^Y-SIRT were included in this study. Uncorrected SPECT images (without attenuation and/or scatter correction) were used as input to a modified 3D shifted-window UNet Transformer (Swin UNETR) architecture. Three separate models were trained to predict attenuation corrected (AC), scatter corrected (SC), and joint attenuation and scatter corrected (ASC) SPECT images. The dataset was split into a training set (~ 80%) and an independent test set (~ 20%). Model training was performed using a five-fold cross-validation framework, with final evaluation conducted on the blind test set. To clinically assess model performance, 3D voxel-wise dosimetry was performed on the test set using the local energy deposition method, assuming ^99m^Tc-MAA as a surrogate for ^90^Y distribution. Quantitative evaluation included organ- and voxel-level metrics, along with Gamma analysis using three combinations of distance-to-agreement (DTA, mm) and dose-difference (DD, %) criteria.

**Results:**

The average (± SD) of the voxel-wise mean error (ME) was ≤ 0.003 Gy for all tasks. The Relative Error (RE (%)) for AC, SC, and ASC tasks were 4.64 ± 7.52%, 8.99 ± 26.35%, and 16.45 ± 25.83%, respectively. Voxel-level Gamma evaluations within the whole body using three different criteria sets, including “DTA: 4.79 mm, DD: 1%”; “DTA: 10 mm, DD: 5%”; and “DTA: 15 mm, DD: 10%” yielded pass rates of over 99.60%. The mean absolute error (MAE) for lesions, normal liver and lungs across all tasks were 3.16 ± 3.39, 0.35 ± 0.36, 0.41 ± 0.47 Gy for AC, 1.97 ± 2.79, 0.19 ± 0.16, 0.22 ± 0.20 Gy, for SC and 5.16 ± 7.10, 0.45 ± 0.51, and 0.34 ± 0.37 Gy for ASC, respectively.

**Conclusion:**

Multiple models were developed for key SPECT quantification tasks, with potential value in clinical setting lacking reliable CT data or sufficient computational resources for Monte Carlo simulations. The models look promising for potential clinical translation and integration into commercial reconstruction software.

**Supplementary Information:**

The online version contains supplementary material available at 10.1007/s12149-025-02152-2.

## Introduction

Selective internal radiation therapy (SIRT) is an established and effective modality for the treatment of both primary and secondary liver malignancies. This locoregional therapy involves the transarterial administration of ^90^Y-labeled microspheres directly into the hepatic arteries to target the tumoral tissue [1, 2]. Prior to treatment, patients routinely undergo planar and SPECT/CT imaging with technetium-99m macroaggregated albumin (^99m^Tc-MAA) to assess any extrahepatic shunting, particularly to the lungs, support patient selection, and enable personalized treatment planning and dosimetry [3, 4]. This simulation is predicated on the assumption that ^99m^Tc-MAA particles replicate the biodistribution of ^90^Y microspheres, lodging within the tumoral microvasculature [5]. Regardless of the controversy over how accurately this surrogate reflects the actual post-therapy dose distribution [6, 7], personalized pre-therapy planning and dosimetry of ^90^Y-SIRT are fundamentally established on this approach [8].

Personalized planning and dosimetry aims to optimize therapeutic efficacy by delivering the highest possible absorbed dose to the tumor while sparing surrounding healthy tissues [9]. This is achieved through individualized pre-therapy dosimetry derived from quantitative SPECT/CT imaging. However, the accuracy of SPECT quantification is affected by several image-degrading physical factors, including photon attenuation and Compton scattering [10, 11]. Accurate attenuation and scatter correction (ASC) is therefore essential, particularly in the context of ^99m^Tc-MAA SPECT imaging, to ensure reliable treatment planning and dose estimation. One study, for instance, has investigated the impact of omitting these corrections on ^99m^Tc-MAA SPECT 3D dosimetry, showing absorbed dose deviations around ± 13% in the absence of attenuation correction and up to + 40% in the absence of scatter correction or jointly ASC compared to fully corrected images [12]. The authors emphasized that treatment planning without scatter correction is not recommended due to its substantial impact on dosimetric accuracy.

Conventional attenuation correction methods employed in clinical practice, based on CT-derived attenuation maps generated through bilinear transformation function, are susceptible to various source of errors [10, 13]. These include inaccuracies in estimating the radionuclide energy or the effective energy of the CT scanner used for attenuation map generation. In addition, CT-related artifacts, such as beam hardening, metal artifacts [14], and motion-induced misalignment between CT and SPECT images [15] can further compromise the accuracy of attenuation correction. Such artifacts may propagate into attenuation-corrected SPECT images, leading to significant biases in activity quantification and consequently in dosimetric calculations.

Several scatter correction methods have been proposed, including model-based, energy window-based, and convolution-based approaches [16–20]. However, each has inherent limitations. For instance, energy window-based techniques, such as the Dual-Energy Window (DEW) and Triple-Energy Window (TEW) methods are prone to down-scatter contamination [21], whereas model-based methods, such as those using Monte Carlo simulations, are computationally intensive and typically require specialized hardware, such as Graphics Processing Units (GPUs) [22].

Inaccuracies arising from the aforementioned factors affect patient-specific dosimetry and treatment planning. Moreover, attenuation correction remains challenging in clinics using standalone SPECT cameras rather than hybrid SPECT/CT systems [23]. In addition, concerns regarding additional radiation exposure from CT scans has highlighted the need to explore alternative approaches [24].

Recent developments in artificial intelligence (AI), particularly in deep learning (DL), have introduced novel strategies for attenuation and scatter correction in both PET and SPECT imaging [25]. A number of DL models have been developed specifically for scatter correction or modeling in ^90^Y SPECT/CT, albeit often trained on relatively small datasets [26, 27]. Our group developed three distinct DL models targeting attenuation, scatter and joint ASC corrections for bremsstrahlung ^90^Y SPECT/CT utilizing a relatively large dataset that underwent extensive clinical validations [28]. These models have been made publicly available. Encouraged by the strong performance of these state-of-the-art architectures, we have extended this work to develop dedicated models for ⁹⁹ᵐTc-MAA SPECT/CT imaging, where to the best of our knowledge, DL-based correction methods have not yet been proposed. What is proposed in the current study is an attempt to improve the accuracy and speed of quantification, which is essential for personalized treatment planning and pre-therapeutic dosimetry in SIRT. Three distinct models were developed, advantaging the accuracy and efficiency of SwinUNETR architecture to perform CT-less attenuation and Monte Carlo-based scatter corrections, both as separate and joint tasks, in ^99m^Tc SPECT/CT imaging.

## Materials and methods

### Patient population

A retrospective study was conducted using SPECT/CT images from a relatively large cohort consisting of 240 patients who underwent ^99m^Tc-MAA imaging prior to ^90^Y-SIRT treatment. All patients were subsequently treated with glass microspheres (TheraSphere™, Boston Scientific, Marlborough, MA, USA) at Geneva University Hospital (HUG, Geneva, Switzerland) between January 2011 and January 2023. Eighteen cases were excluded due to CT artifacts and/or technical issues during image processing, resulting in a final dataset of 222 patients (172 males, 50 females), with a median age of 67 years (SD ± 13.35, range 26–92). The average ± SD of body weight and height of the patients were 75.80 ± 18.39 kg and 1.71 ± 0.10 m, respectively. Since optimal deep learning training requires high-quality, artifact-free data, only cases with reliable CT image quality were included to ensure that the model learned attenuation and scatter related patterns rather than artifact-induced distortions. Accordingly, 18 cases presenting with severe CT artifacts, such as metal, respiratory motion, streak (including truncation), and ring artifacts were excluded. Technical exclusions also included incomplete or corrupted data, reconstruction failures, and uncorrectable CT-SPECT misregistration during preprocessing.

### SPECT/CT acquisition and reconstruction

For ^99m^Tc-MAA simulation, the patients received an average ± SD intra-arterial injected activity of 158.82 ± 34.68 MBq. Planar and SPECT/CT imaging was performed on Symbia-T16 or T6 cameras (Siemens Healthineers, Erlangen, Germany). SPECT acquisitions were conducted with a low-energy, high-resolution (LEHR) collimator and a 140 keV energy window (± 15% width), using 64 projections over 360° arc (20–25 s per projection), in a 128 × 128 matrix with a voxel size of 4.795 mm. SPECT images were reconstructed using Hermes HybridRecon™ (Hermia Reconstruction) version 4.0 (Hermes Medical Solutions Ltd, Stockholm, Sweden), calibrated for ^99m^Tc. Hermia reconstruction uses a modified Ordered Subsets-Expectation Maximization (OSEM) algorithm that incorporates CT-based attenuation correction, GPU-based Monte Carlo simulation for scatter correction, and pre-calculated look-up tables to compensate for collimator and detector response [29–32]. All patient data were reconstructed using 4 iterations and 8 subsets, followed by a Gaussian post reconstruction filter of 5 mm FWHM, which was the clinical routine for this examination. SPECT images were reconstructed three times: (i) uncorrected images (no attenuation and scatter correction, NC), (ii) only attenuation correction (AC), and (iii) attenuation and scatter correction (ASC). Co-registered CT scans were acquired using tube voltages of 110 kVp (191 cases) and 130 kVp (31 cases), with an average ± SD tube current of 51.6 ± 21.6 mA. CT images were reconstructed in a 512 × 512 matrix with a pixel size of 0.977 × 0.977 mm^2^, and slice thickness of 3 mm.

### Preprocessing and network training

The original SPECT images were normalized such that the minimum voxel value was set to zero and the maximum was set to the 99th percentile of each image’s voxel values, without clipping. To mitigate overfitting, random patch sampling was applied, involving cropping or padding of different regions. During training, multiple patches of size 96 × 96 × 96 voxels were extracted from each normalized SPECT image. These patches were input to a modified 3D shifted window UNet Transformer (Swin UNETR, version 2) model [33], using a batch size of 2 and the original isotropic voxel spacing of 4.795 mm³. This architecture was previously employed in our earlier work on ^90^Y SPECT quantification. A detailed rationale for selecting Swin UNETR and its implementation for these correction tasks is provided in [28].

In this study, the default activation function of the Swin UNETR (“Leaky ReLU”) was manually replaced with “ReLU” to avoid negative output values. Weight and bias values were initialized from an identity weight to ensure that initial outputs closely resembled the inputs. Data augmentation included only random rotations and flips. Deformable augmentations were intentionally excluded to maintain the quantitative integrity of the data. To reduce order bias, the training data were shuffled prior to training.

Patient data were split into 80% (178 cases) for training and 20% (44 cases) as a blind test set. The training set was further divided into 5 folds to be used in a 5-fold cross-validation framework, where 80% of the training data (~ 142 cases) was used for internal training and 20% (~ 36 cases) for validation. Five models were trained independently on these folds, and their predictions were ensembled by averaging their outputs in the external test set, which remained blind during training.

Model training was conducted using 3D patches of 96 × 96 × 96 voxels, with a voxel size of 4.795 mm^3^. The networks were trained for 100 epochs using the Adam optimizer and an initial learning rate of 1 × 10^− 3^, a weight decay of 1 × 10^− 4^, and a batch size of 2. The L1 loss (Mean Absolute Error) was used as the loss function. All training and inference were conducted using PyTorch (version 2.7) within the Medical Open Network for Artificial Intelligence (MONAI) framework (version 1.4) on an NVIDIA GeForce RTX 4090 GPU with 24 GB of VRAM. Inference on test images was performed using a sliding window strategy with 50% patch overlap.

Three different models were trained for three different tasks, namely:

1. AC task, where AC SPECT was predicted from NC SPECT;
2. SC task, where ASC SPECT was predicted from AC SPECT;
3. ASC task, where ASC SPECT was predicted from NC SPECT.

The input and output for each model are specified in Table 1.

**Table 1:** Input and reference image pairs used for training in “AC”, “SC”, and “ASC” tasks.

Task	Model Input	Model Output (reference)
AC	NC SPECT	CT-based AC SPECT
SC*	AC SPECT	ASC SPECT
ASC	NC SPECT	ASC SPECT

*Note: in the SC task, ASC images serve as Monte Carlo-based scatter correction references, with AC SPECT as input

### Quantitative evaluations

To quantitatively and clinically evaluate model performance, organ-level and 3D voxel-wise dosimetry were performed on the test set using the local energy deposition method (LDM) across NC, AC, ASC, and DL-generated images. The analysis followed the following steps:

- Segmentation
  The lesions were manually segmented on diagnostic contrast-enhanced CT (CECT) or MR images by an experienced nuclear medicine physician. The whole liver (WL) and Lung structures were delineated automatically on co-registered CT images from ^99m^Tc-MAA SPECT/CT images using previously trained and publicly available deep learning networks dedicated to organ segmentation developed in our group [34, 35] followed by visual checking and modification if necessary. Then, the whole normal liver (WNL) was obtained by subtracting the tumor segmentations from WL segmentation.

b) Image registration
  The CECT or MRI images were registered on the co-registered CT from ^99m^TC-MAA SPECT/CT images through rigid registration with the Mutual information cost function in elastix platform to transfer lesion segmentations on ^99m^Tc-MAA SPECT/CT images. Registration accuracy was carefully assessed through visual inspection by experienced physicians, focusing on the alignment of anatomical landmarks, such as liver and lung boundaries.

c) Dose calculation
  3D voxel-level dosimetry based on LDM was performed. Indeed, the dose calculations were implemented as if the ^99m^Tc-MAA particles act as a surrogate of ^90^Y microspheres. The assumption in LDM is that every beta emission energy is deposited in the occurring voxel, and there are no cross-talks amongst voxels (Eq. 1).

1 $$\:{D}_{\mathrm{v}\mathrm{o}{\mathrm{x}}_{\mathrm{t}}}\left(x\right)={\left.{\stackrel{\sim}{A}}_{\mathrm{v}\mathrm{o}{\mathrm{x}}_{\mathrm{s}}}\left(x\right)\times\:S\left(\mathrm{v}\mathrm{o}{\mathrm{x}}_{t}\leftarrow\:\mathrm{v}\mathrm{o}{\mathrm{x}}_{s}\right)\right|}_{t=s}$$

The half-life ($\:{T}_{1/2})\:$of ^90^Y was set to 64.1 h, with a mean beta energy of 0.93 MeV, and a liver density of 1.05 g/cm^3^. Patient-specific calibration was applied, wherein we considered all counts inside the whole liver volume to convert the SPECT images (in counts/sec) to quantitative images (in Bq/ml). Dosimetry calculations were performed using an in-house Python code. The dosimetry calculation details are provided in our previous studies [28, 36–38].

**Fig. 1:**
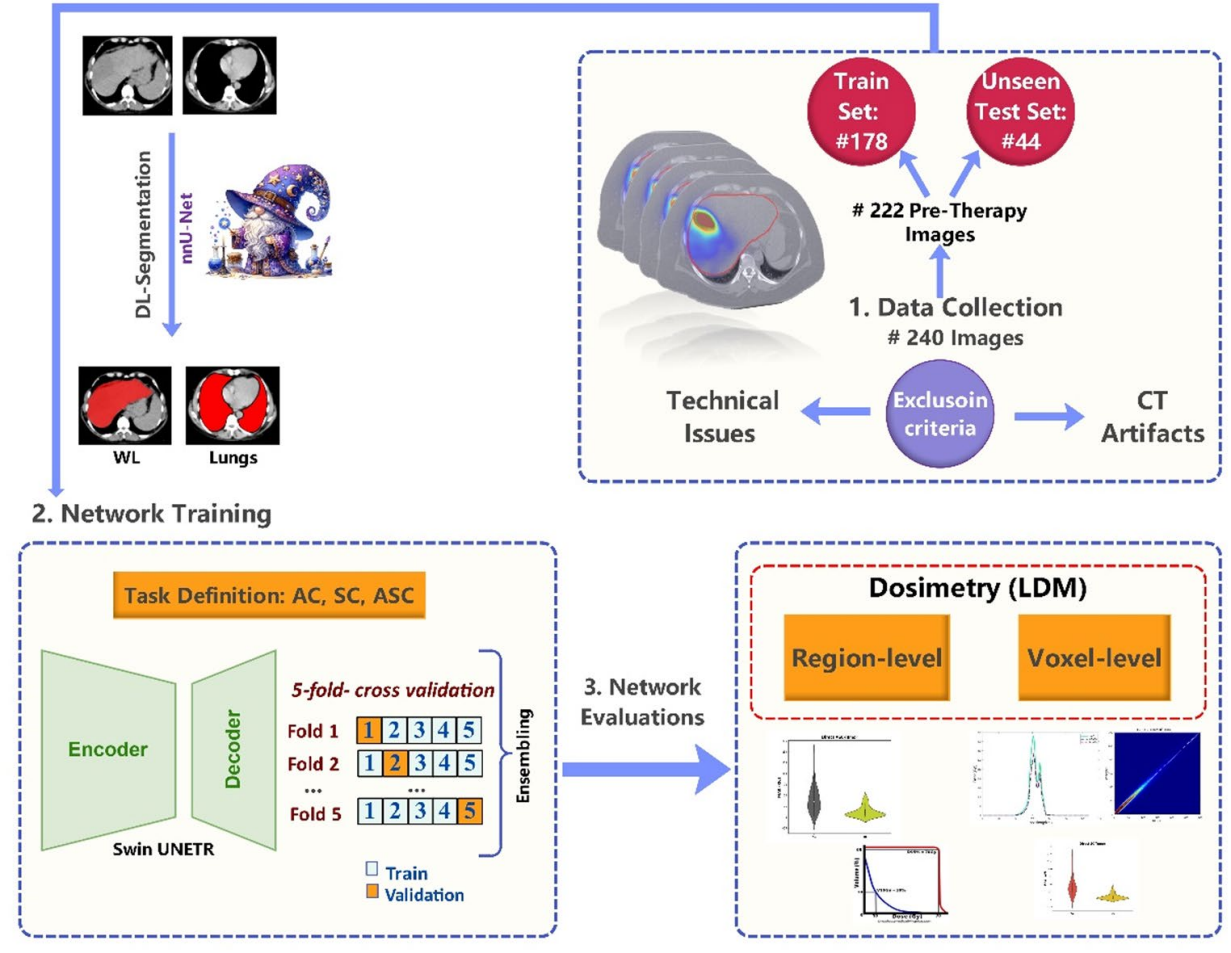
Flowchart of this study showing all the considered steps. (1) The dataset consisting of 240 cases was collected and after excluding 18 cases due to technical issues and CT artifacts, the remaining data were split into train and test sets. Key organs were delineated using DL-based segmentation models. (2) the networks were trained for three different tasks using Swin UNETR and 5-fold cross-validation. (3) the model performance was evaluated quantitatively using voxel and region-wise dosimetry metrics

d) Analysis
  The dosimetry results from model inputs and outputs were compared to reference both voxel-wise and region-wise. Voxel-wise evaluations included calculating quantitative metrics, such as structural similarity index (SSIM), root mean squared error (RMSE), peak signal-to-noise ratio (PSNR), mean absolute error (MAE), mean error (ME), relative error (RE), and relative absolute error (RAE). The image level voxel-wise evaluation was performed using only voxels inside the patients’ body contour. These metrics were compared across DL-results, uncorrected (input) and reference dose maps using the Mann-Whitney U-test. In addition, analysis such as joint histograms, dose map line profiles, and 3D gamma evaluation [39] between reference and DL results were conducted. The distance to agreement/dose difference (DTA/DD) criteria for gamma analysis included three different sets: 4.795 mm /1%, 5% and 10 mm, and 10% and 15 mm to explore suitable criteria for gamma evaluations in SIRT.
  The region-level analysis was conducted for three key regions including lesions, WNL, and lungs. Quantitative metrics, such as MAE, ME, RE, RAE and median shift were calculated for each. Dose-volume histograms (DVHs) were also calculated for these regions. The mean absorbed dose (MAD) was compared across DL results, uncorrected, and reference images using Mann-Whitney U-test. The flowchart of this study is presented in Fig. 1.

## Results

### Voxel-level evaluations

**Fig. 2:**
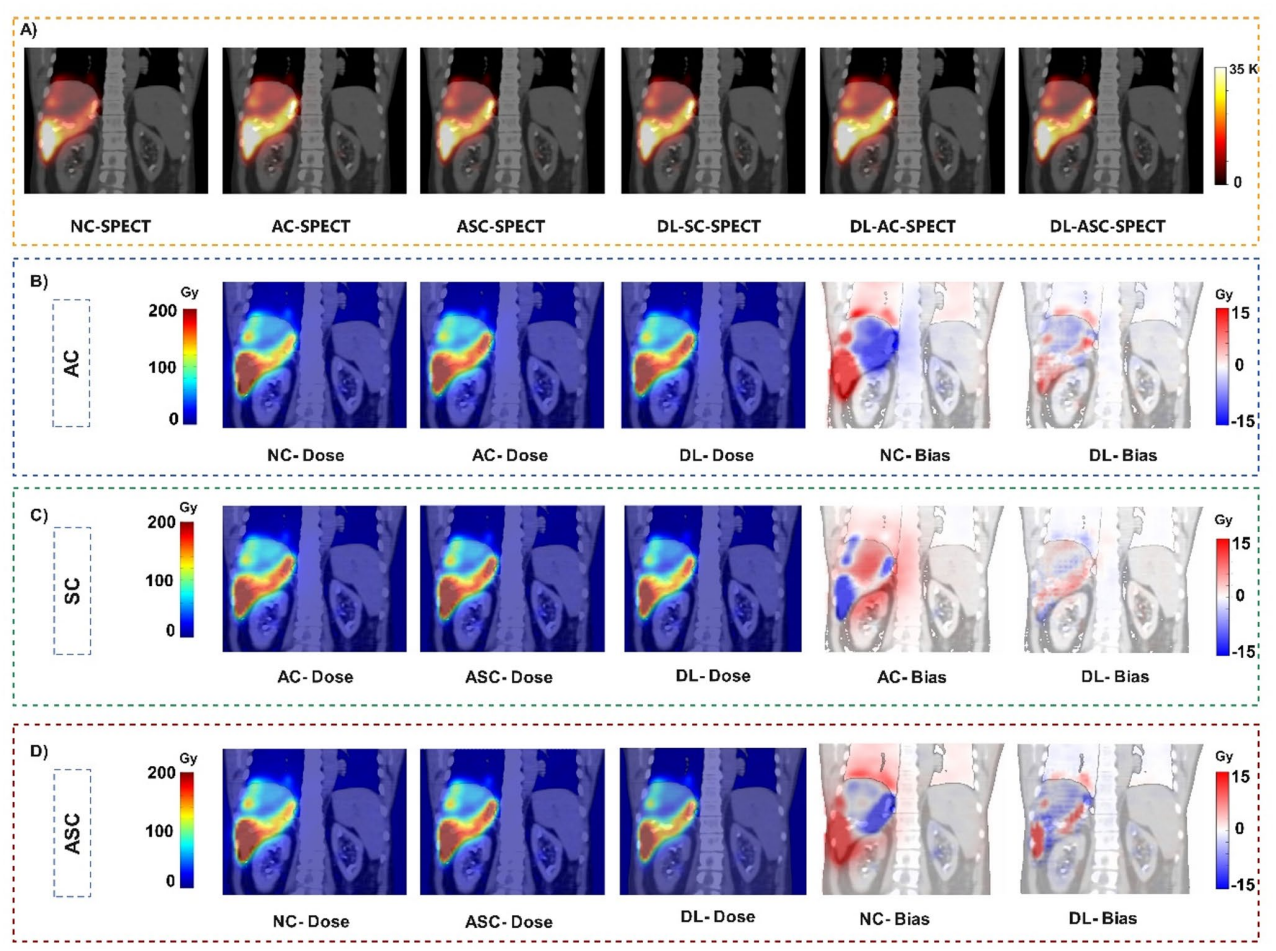
Representative images in coronal view illustrating the input, reference, and DL-predicted outputs across all correction tasks. (**A**) SPECT images from uncorrected (NC), attenuation corrected (AC), and attenuation + scatter corrected (ASC) inputs, alongside DL results for each corresponding task. B–D) Dose maps for the (**B**) AC, (**C**) SC, and (**D**) ASC tasks are shown, including input, reference, and DL-predicted results. Corresponding DL bias maps demonstrate superiority of the results compared to input bias maps. The negative values in the colorbars of the bias maps indicate regions where the reference dose map has higher values than the predicted dose map and does not mean the presence of negative dose values in the results

Representative NC, AC, ASC SPECT images, and the results of DL models for each task, overlaid on CT images along with the dose maps calculated on input, DL outputs and standard of reference are illustrated in Figure 2. In addition, the bias maps with respect to the reference dose maps for both input and DL results are shown. All images correspond to one patient (#210), for which the model’s performance was close to the average of the reported results. The bias maps show the superiority of DL results over the inputs of the model in the tumoral and normal liver as well as the lungs. In AC and ASC tasks, the regions in the body surfaces, tumoral region, and within the lungs are overdosed in the NC-bias maps. whereas the errors are mitigated in these regions as shown in the DL bias maps.

**Fig. 3:**
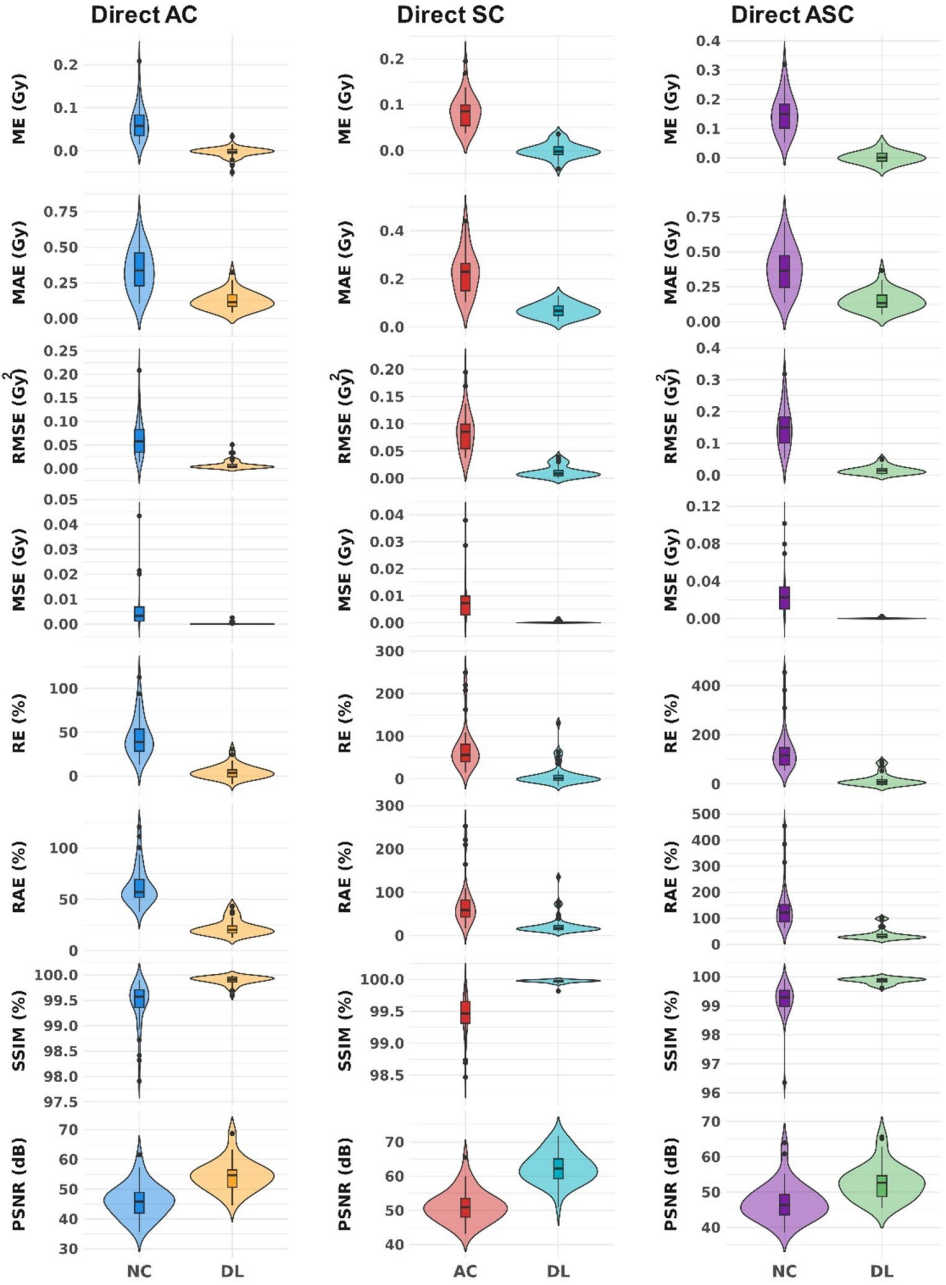
Violin plots showing the distribution of quantitative metrics for voxel-level evaluations, displayed from top to bottom as follows: SSIM (%), PSNR (dB), MAE (Gy), MSE (Gy), RMSE (Gy), RAE (%), and RE (%). Metrics from left to right are shown for AC, SC, and ASC tasks. The p-values from Mann-Whitney U-test comparing the metrics between input and DL results were < 0.05, showing significant differences between them

The average ± SD of the voxel-level quantitative metrics for AC, SC and ASC tasks are summarized in Table 2. Moreover, the distribution of these metrics is shown in the violin plots in Fig. 3. The p-values from Mann-Whitney U-test comparing the metrics between input and DL results were < 0.05, demonstrating statistically significant differences between them.

**Table 2:** Average ± SD of voxel-level quantitative metrics across all tasks

	SSIM (%)	PSNR (dB)	ME (Gy)	MAE (Gy)	RMSE(Gy^2^)	MSE(Gy)	RE (%)	RAE (%)
AC	99.89 ± 0.08	54.52 ± 5.04	-0.003± 0.012	0.122 ± 0.060	0.008±0.010	0.0001 ± 0.0004	4.64 ± 7.57	22.53 ± 7.77
SC	99.96 ± 0.02	62.65 ± 4.41	-0.00022 ±0.01618	0.068 ± 0.027	0.012 ± 0.010	0.00025 ± 0.00039	8.99 ± 26.35	24.77± 23.33
ASC	99.86 ±0.097	52.51 ± 4.72	0.00098 ± 0.01981	0.148 ± 0.067	0.015 ± 0.012	0.00038 ± 0.00058	16.45± 25.83	37.66 ± 22.34

**Fig. 4:**
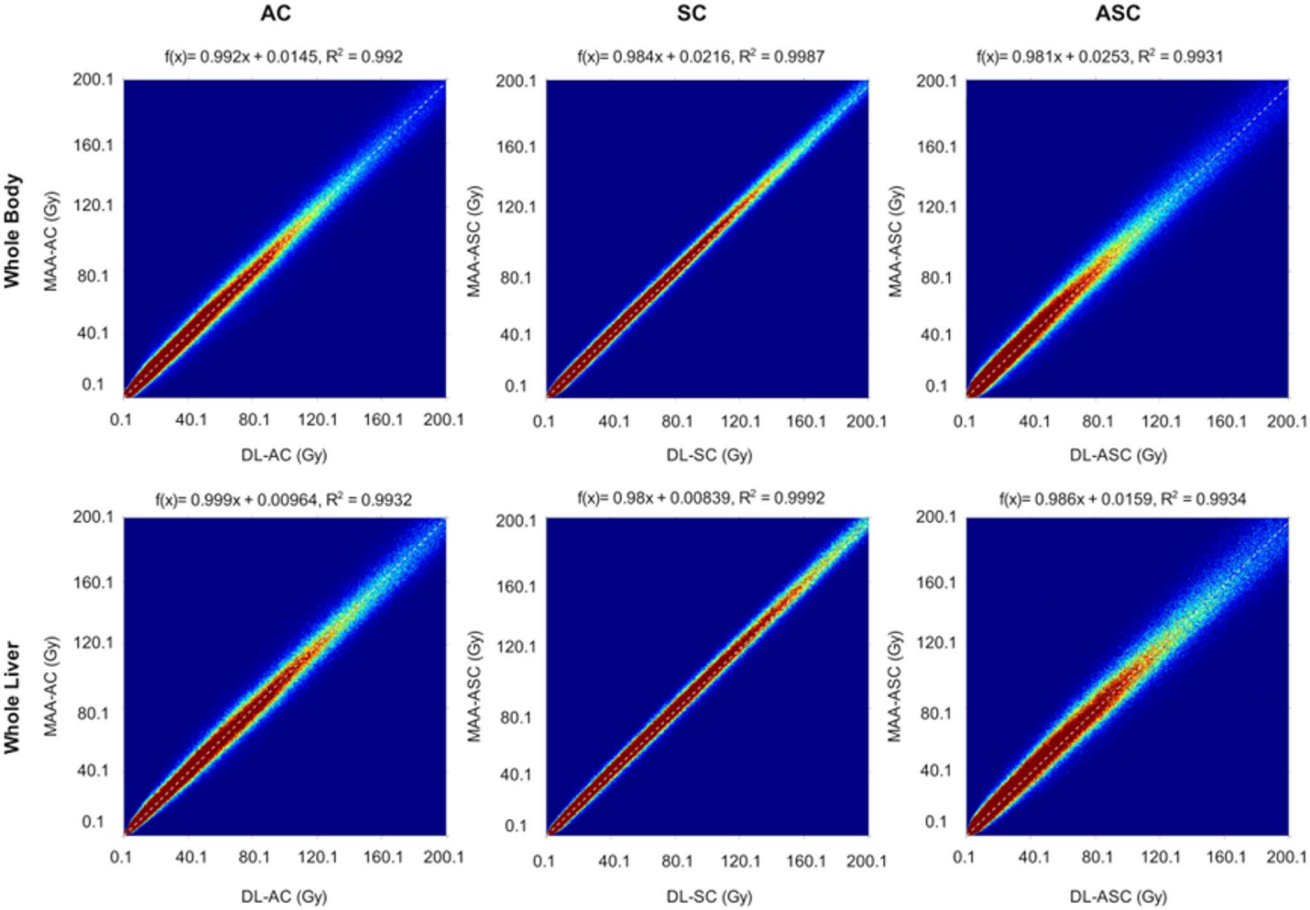
The joint histograms comparing reference dose maps and DL dose maps for each task is shown, calculated for the whole image (top) and for the whole liver (bottom)

The joint histograms were calculated to compare the dose maps from DL results and standard of reference of each task. These histograms calculated once within the whole body and once within the whole liver are shown in Fig. 4. The visual results with R^2^ of more than 0.99 demonstrate a strong correlation between the reference and DL dose maps both in whole body and in the whole liver.

**Fig. 5:**
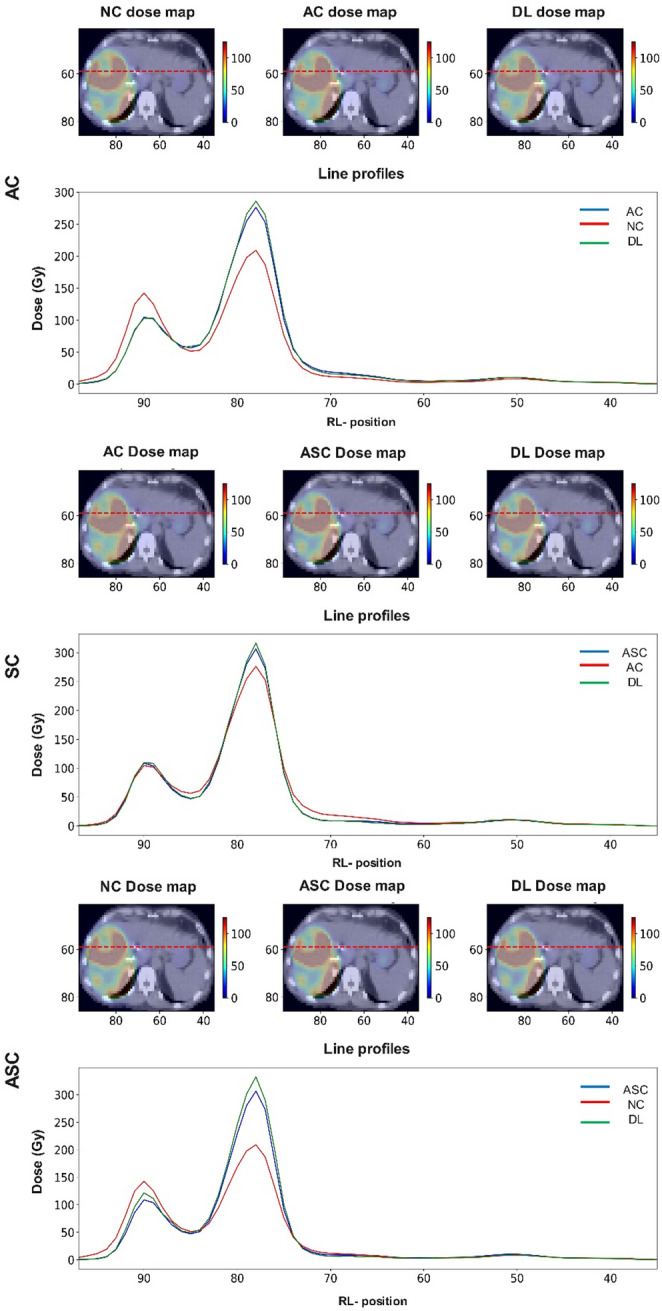
The line profiles illustrate strong agreement between the DL predictions and the reference, with noticeable deviations observed in the input. The profiles are extracted from the same axial slice and follow the right-to-left (RL) direction. The input, reference, and DL outputs are shown in red, blue, and green, respectively. Top panels display fused CT and dose maps, whereas the color bars indicate dose intensity values

Figure 5 depicts the axial (right-to-left) line profiles comparing the input, DL, and reference dose maps. The line-profiles are from the same axial slice crossing the tumor of a patient. The figure shows excellent alignment between the green (DL) and the blue lines (reference), across the whole axial plane (within the tumoral and non-tumoral regions). The best agreements are observed for SC and then AC tasks. Table 3 summarizes the pass rates (%) from gamma evaluation with three different criteria, calculated within the whole image, tumor, WNL, and lungs. The corresponding values of pass rates comparing the inputs and reference for each task are provided in Supplementary Table 1.

**Table 3:** Average gamma pass rates for each task, evaluated across three distance-to-agreement (DTA, mm) and dose difference (DD, %) criteria. Results are reported for the whole body, tumor, whole normal liver (WNL), and lung regions

DTA & DD	4.795 mm & 1%			10 mm & 5%			15 mm & 10%		
	**AC**	**SC**	**ASC**	**AC**	**SC**	**ASC**	**AC**	**SC**	**ASC**
Whole image	99.67 ±0.19	99.78 ± 0.15	99.67 ± 0.20	99.97 ±0.02	99.99 ±0.01	99.97 ±0.02	99.99 ±0.00	100 ±0.00	99.99 ±0.00
Tumors	97.57 ±1.42	97.88 ±1.45	97.56 ±1.40	99.60 ±0.70	99.92 ±0.14	99.61 ±0.65	99.96 ±0.08	100 ±0.0	99.94 ±0.20
WNL	98.38 ±0.67	98.71 ±0.63	98.31 ± 0.71	99.76 ±0.24	99.96 ±0.03	99.75 ±0.24	99.97 ±0.03	100 ±0.0	99.97 ±0.04
Lungs	87.40 ±9.14	93.15 ±6.53	89.05 ± 9.94	99.35 ±1.40	99.82 ±0.60	99.55 ±0.96	99.90 ±0.36	100 ±0.002	99.93 ± 0.24

### Region-level evaluations

**Fig. 6:**
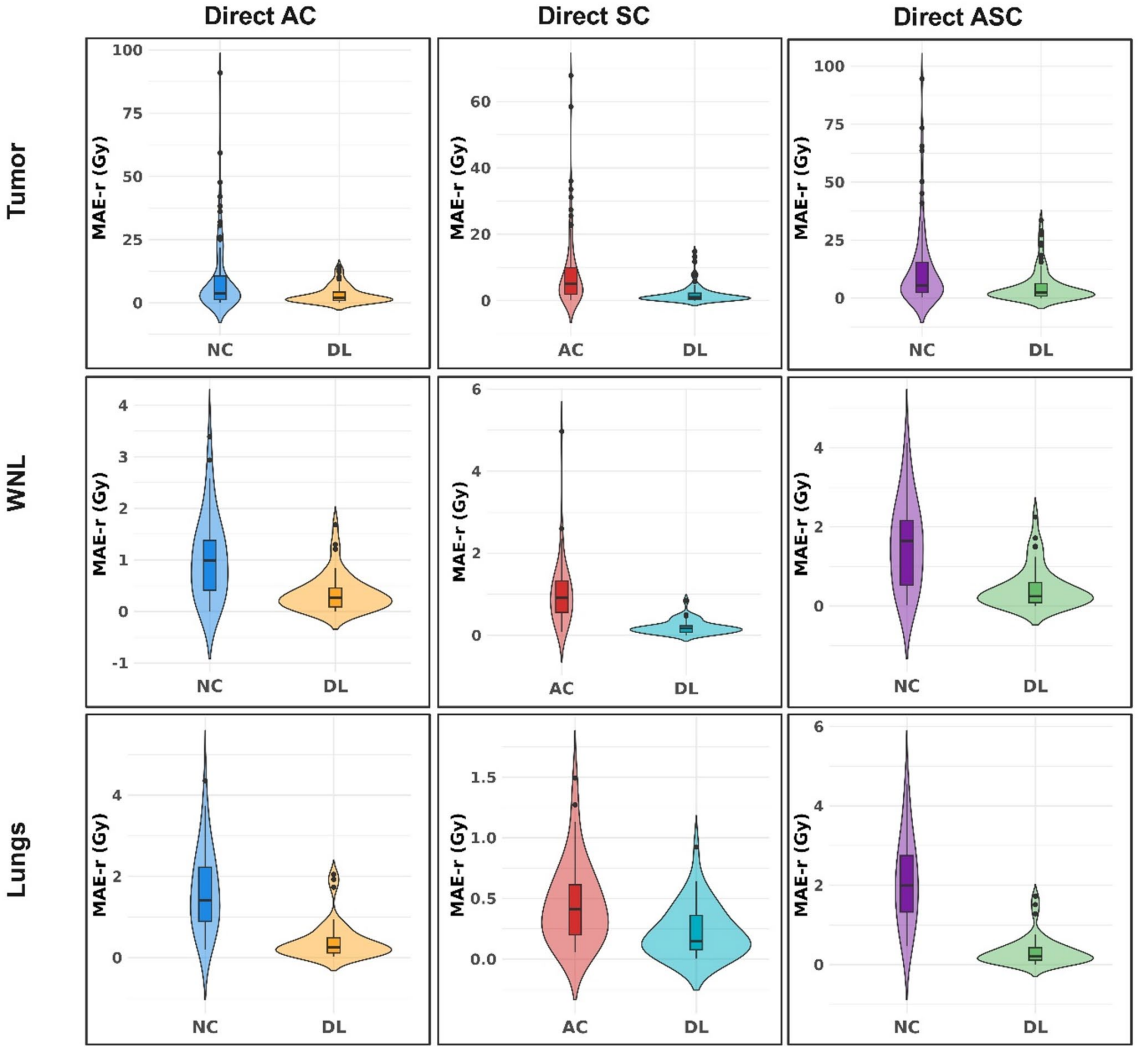
The distribution of the region-level mean absolute errors comparing the errors between inputs and reference vs. DL and reference, for all three tasks

The distribution of the organ-level mean absolute errors (MAE) for tumors, WNL, and lungs is illustrated in Fig. 6. These violin plots show how organ errors with respect to reference image decrease when using our DL models for each task compared to the inputs. To further improve clarity, the plots for other error metrics, such as ME, RAE, and RE, are provided in Supplemental Figs. 1–3 for AC, SC, and ASC tasks, respectively. The mean ± SD of the MAE (Gy) for AC task is 3.16 ± 3.4, 0.35 ± 0.36, and 0.41 ± 0.47 Gy for tumors, WNL, and lungs, respectively. These values were 1.97 ± 2.79, 0.19 ± 0.16, and 0.22 ± 0.20 Gy for the SC task, respectively, whereas they are for ASC 5.16 ± 7.10, 0.45 ± 0.51, and 0.34 ± 0.37 Gy, respectively. These values showed significant improvement compared to the input images as shown in Fig. 6. The organ-level errors are summarized in Table 4.

**Table 4:** Average ± SD of organ-level quantitative metrics across all tasks

Organ	Task	RE%	RAE%	ME(Gy)	MAE(Gy)	Median Shift (Gy)
Tumors	**AC**	-0.03 ± 9.31	5.64 ± 7.37	0.033 ± 4.656	3.16 ± 3.39	-0.34 ± 4.25
	**SC**	2.42 ± 16.7	7.08 ± 15.30	0.53 ± 3.39	1.97 ± 2.79	0.73 ± 3.11
	**ASC**	0.52 ± 15.36	8.16 ± 12.99	-0.06 ± 8.8	5.16 ± 7.10	-0.21 ± 8.08
WNL	**AC**	-0.791 ± 1.24	0.99 ± 1.08	-0.27± 0.43	0.35± 0.36	-0.48 ± 1.23
	**SC**	0.26 ± 1.16	0.58± 1.03	0.09± 0.24	0.19 ± 0.16	-0.08 ± 0.65
	**ASC**	-0.52 ± 2.6	1.45 ± 2.21	-0.143 ± 0.674	0.45 ± 0.51	-0.86 ± 1.6
Lungs	**AC**	11.91 ± 15.04	14.76 ± 12.18	0.32 ± 0.53	0.41 ± 0.47	0.11 ± 0.25
	**SC**	-6.42 ± 8.11	8.80 ± 5.36	-0.19± 0.22	0.22 ± 0.20	-0.13 ± 0.15
	**ASC**	13.99 ± 19.55	17.05 ± 16.88	0.27 ± 0.42	0.34 ± 0.37	0.08 ± 0.22

**Fig. 7:**
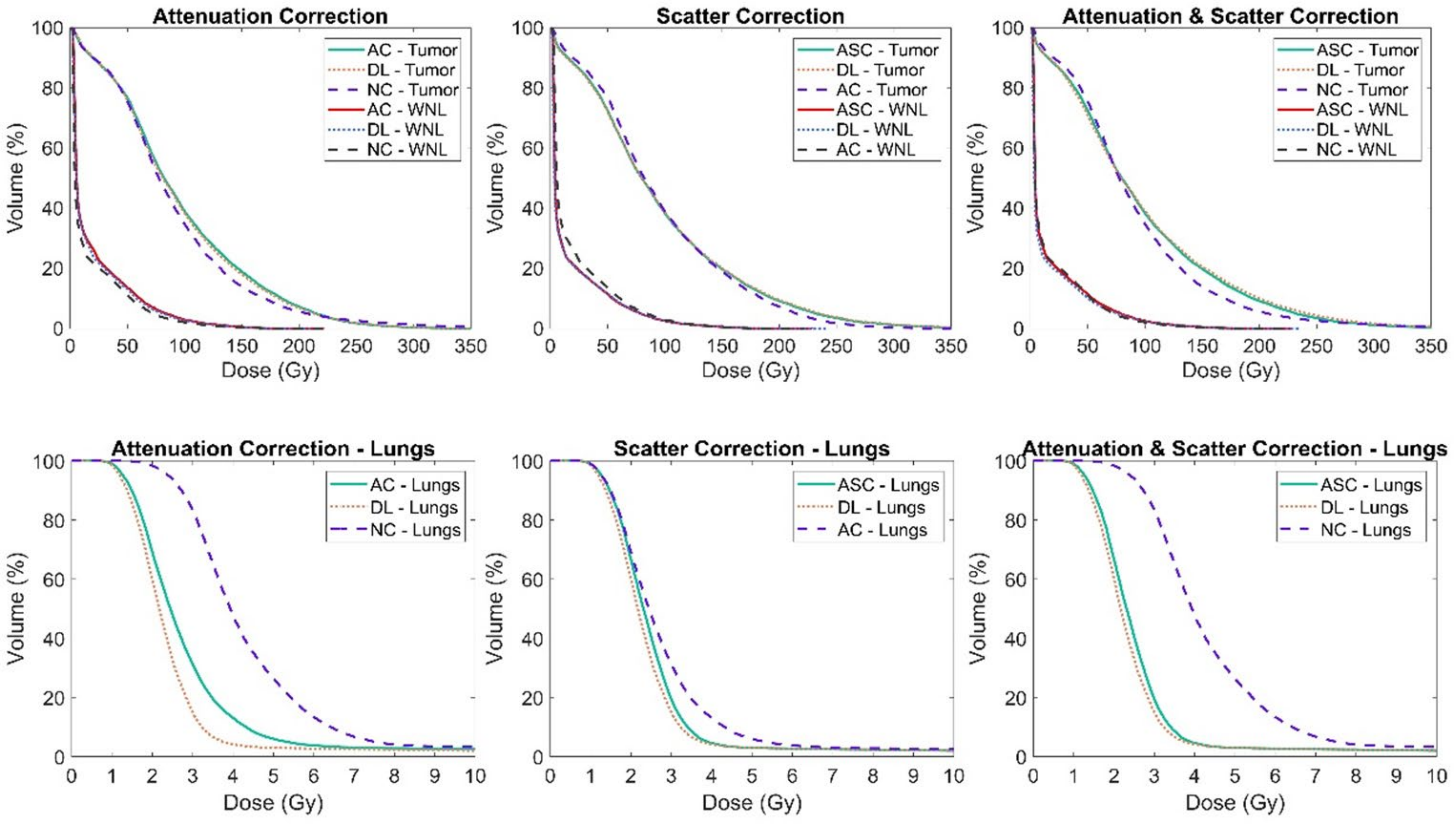
Plots of dose volume histograms for tumors and WNL (top) and for lungs (bottom) derived from the input, reference and DL-based results of each task

The mean absorbed dose (MAD) ± standard deviation (SD) for each organ, calculated from the reference, input, and DL-predicted images, along with the p-values from Mann-Whitney U tests comparing input and DL results to the reference MAD values, are presented in Supplemental-Table 2. We also calculated the DVH for tumors, WNL, and lungs, which are shown in Fig. 7.

## Discussion

In this study, three distinct deep learning models were developed to generate attenuation and scatter corrected ^99m^Tc-MAA SPECT images, either as separate or joint tasks, from non-attenuated and/or non-scatter-corrected inputs, utilizing Monte Carlo scatter correction without relying on transmission images. A key strength of this study is the use of a relatively large training dataset, as well as extensive performance evaluation through voxel- and organ-wise dosimetry. To the best of our knowledge, this is the first study to train DL models aiming at enhancing and accelerating quantification of ^99m^Tc-MAA SPECT images for accurate and personalized SIRT treatment planning and pre-therapy dosimetry. The performance evaluation and quantitative metrics reported were all in the dosimetry domain based on the local energy deposition method as we believe the absorbed dose metrics are more meaningful and consistent than SPECT tracer uptake estimates, consisting of less relevant count-based values [28]. It should be emphasized that dosimetry using LDM is essentially a scaled representation of SPECT images via a patient-specific calibration factor. Therefore, the quantitative metrics calculated in the dose domain are effectively representative of the underlying SPECT image quality, yet more interpretable and clinically meaningful.

It has been recommended that, when CT-based attenuation correction is not feasible, non-attenuated-corrected images may still be used for estimating mean absorbed doses in lesions and whole normal liver, as attenuation effects can be partially compensated in LDM-based dosimetry [12, 40, 41]. However, performing dosimetry without scatter correction is not advised [12]. It is well known that even attenuation- and scatter-corrected ⁹⁹ᵐTc-MAA SPECT may slightly over/underestimate absorbed dose to tumor or normal liver when compared to actual dose values with ⁹⁰Y PET/CT, limitations that can be more exacerbated in uncorrected images. Therefore, we developed deep learning models capable of generating corrected images in scenarios where attenuation and/or scatter correction is not possible, to support accurate and personalized treatment planning for SIRT. Our results demonstrate that dosimetry based on DL-corrected images are much more reliable than those with non-corrected approaches. This study therefore makes an important step toward improving the accuracy and efficiency of ^90^Y-SIRT treatment planning and personalized therapy, with potential to support clinical adoption and improve outcomes in personalized radionuclide therapy.

MC-based attenuation and scatter correction is widely regarded as the most accurate method for modeling these effects. However, its routine clinical application is often limited by the significant computational demands. In this study, we used the Hermes SPECT reconstruction tool which models scatter effects based on GPU-based MC simulation to generate reference images. Our primary objective in training these models was to overcome the computational burden associated with Monte Carlo simulations, and to provide a solution for centers without access to GPU-based correction tools. While HybridRecon™ is highly efficient on GPUs, running the same correction on a CPU can take over an hour, up to 80 min per scan. In contrast, our deep learning models produced results in about 4 s per image on a GPU, and approximately less than one minute on a standard Intel Core i9-13900KF CPU, a much more practical alternative for routine clinical use.

Additionally, attenuation correction using our proposed DL models is performed directly without the need for CT scans, making it a practical solution for centers that rely on standalone SPECT systems or aiming at reducing radiation dose to patients. During visual inspection, we excluded cases with CT artifacts to prevent training the models on data where artifacts may have propagated to CT-based reference images. This careful image selection and data cleaning allowed to improve the accuracy of attenuation correction by training the models on a clean, artifact-free dataset with Monte Carlo-based scatter correction.

In this study, we used the Swin UNETR architecture, a state-of-the-art DL model, motivated by its strong performance in our previous work on ^90^Y- SPECT quantification [28]. While ^90^Y-SPECT is considerably noisier, we expected the model to perform even better on ⁹⁹ᵐTc-MAA SPECT, and our results confirmed this assumption. The architecture is a hybrid of UNet-like encoder-decoder structure and transformer-based attention, which can capture fine and global features simultaneously.

In this study, the models were trained to perform AC, SC, and combined ASC as separate and joint tasks, showing promising results across all approaches. From a voxel-wise perspective, as illustrated in Fig. 2 the bias maps indicate that, in the SC task, the absence of scatter correction (represented by the AC bias map) leads to absorbed dose overestimation in the WNL. Similarly, in AC and ASC tasks, the NC bias maps highlight overestimation in high-uptake regions, such as the tumor, when both corrections are missing. These observations are consistent with the findings reported by Botta et al. [12]. Organ-wise analysis showed that In ASC and AC tasks (Supplemental Tables 1 and 2), a significant statistical difference is observed in “lung absorbed dose”, which is of importance when calculating lung shunt fraction pre-therapy. All errors voxel and region -wise were mitigated when applying our DL models.

The three proposed models address complementary clinical scenarios. AC and SC models enable correcting for attenuation and scatter effects and may be useful in situations where only partial correction is available or for methodological evaluation of their individual contributions to dosimetry. The ASC model represents the most clinically relevant approach, especially for SPECT-only systems where neither CT-based attenuation correction nor model-based scatter correction can be applied. However, joint correction for attenuation and scatter constitutes a more challenging task, as the model needs to learn both patterns simultaneously which may explain the slightly reduced performance of ASC compared with single-task models (AC and SC). Nevertheless, ASC model provided improved performance relative to uncorrected images.

The dataset used in this study largely overlaps with that of our previous work on ^90^Y-SIRT quantification [28], therefore models are similarly sensitive to high-density objects (e.g., titanium spinal implant), and heterogeneous tissue compositions (e.g., calcifications, tumor adjacent to lung tissue). These cases (patients with metal implants or tumors located near tissue interfaces) were limited in our training dataset, and are more challenging for the joint ASC task, as attenuation and scatter effects must be corrected simultaneously within the same regions. These scenarios were associated with larger voxel-level deviations compared with anatomically more homogeneous cases. While DL-ASC performed well in most cases, careful interpretation is recommended for such anatomically heterogeneous scenarios, particularly in SPECT-only settings where CT-based verification is unavailable.

We conducted comprehensive evaluations of the models using a separate test dataset that was not exposed during training. These evaluations include region-wise and voxel-wise clinically meaningful dosimetry analyses to highlight the strengths and limitations of each model, so the end-users would be able to make informed decisions about model applicability, reliability, and integration into clinical workflows. However, this study has several limitations. The datasets were acquired from a single center using one scanner, which may limit the generalizability of the models. To address this, future work will involve training on larger, multi-center datasets collected using different scanners and imaging protocols. Once generalizability is confirmed and the models validated on external datasets, clinical deployment will become more feasible. Additionally, since the models support both separate and joint attenuation and scatter correction tasks, they have the potential to be integrated with commercial reconstruction software. The trained models and detailed inference instructions will be made publicly available on our lab’s GitHub page following publication.

## Conclusion

The models were developed to perform CT-free attenuation correction and/or Monte Carlo-based scatter correction of ^99m^Tc-MAA SPECT images, either as separate or joint tasks. A relatively large dataset was reconstructed using the Hermes Hybrid Recon™ tool to serve as standard of reference for model training and evaluation. Model performance was extensively assessed through region-wise and voxel-wise dosimetric analyses to support personalized treatment planning in SIRT. Given the promising results, these models are potentially clinically useful, especially in centers equipped with standalone SPECT systems or those lacking computational resources for MC-based corrections. The models could also be integrated into commercial reconstruction software. These applications would be possible after further training on larger, and more diverse datasets.

## Supplementary Information

Below is the link to the electronic supplementary material.

Supplementary Material 1

## Data Availability

The data used in this work are not available. These models will be made available on our group Github (https://github.com/pinlab-group) and (https://github.com/ZahraMansouriMedPhys).

## Abbreviations

AC: Attenuation correction
ASC: Attenuation and scatter correction
DD: Dose-difference
DEW: Dual energy window
DL: Deep learning
DTA: Distance-to-agreement
DVH: Dose-volume histograms
GPU: Graphics processing units
MAA: Macroaggregated albumin
MAD: Mean absorbed dose
MAE: Mean absolute error
MC: Monte Carlo
ME: Mean error
NC: No attenuation and scatter correction
OSEM: Ordered Subsets Expectation Maximization
PET: Positron emission tomography
PSNR: Peak signal-to-noise ratio
RAE: Relative absolute error
RE: Relative error
RMSE: Root mean squared error
SC: Scatter correction
SD: Standard deviation
SIRT: Selective internal radiation therapy
SPECT: Single photon emission tomography
SSIM: Structural similarity index
TEW: Triple energy window
WL: Whole liver
WNL: Whole normal liverDL: deep learning
AI: Artificial intelligence
RAM: Random Access Memory
LEHR: Low-energy, high-resolution
Swin UNETR: Shifted window UNet TRansformer
LDM: Local deposition method
CECT: Contrast enhanced computed tomography
WL: Whole liver
WNL: Whole normal liver
