# Supplementary material for: Deep learning-guided attenuation and scatter correction of 99mTc-MAA SPECT images: towards quantitative analysis in 90Y-SIRT
Source: Ann Nucl Med. 2026 Jan 5;40(5):507–20. doi: 10.1007/s12149-025-02152-2 (PMC13124837; doi:10.1007/s12149-025-02152-2)
Supplement: Supplementary file 1 — Supplementary Material 1 [file 12149_2025_2152_MOESM1_ESM.pdf]

Supplementary material

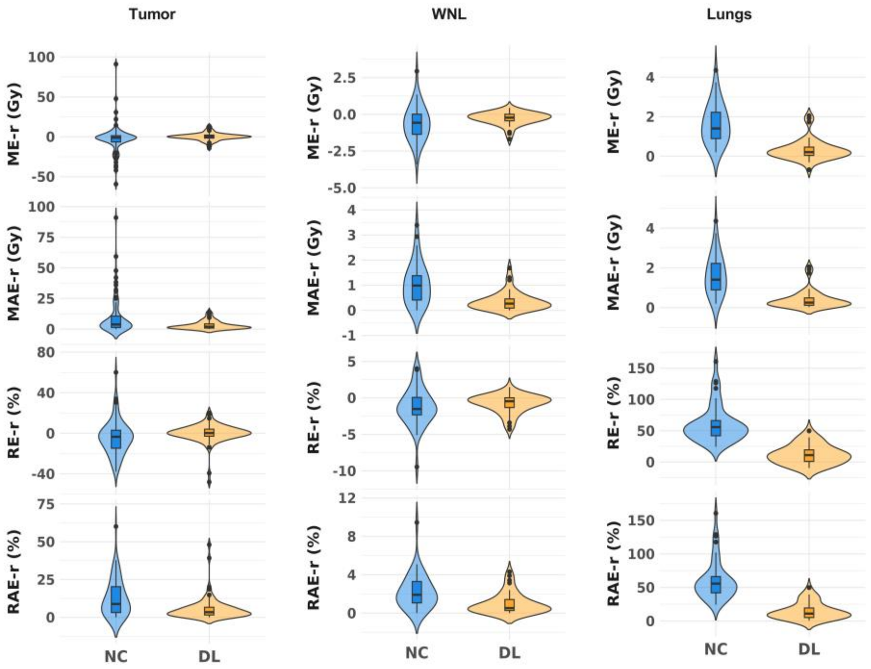

Supplemental-Figure 1. The distribution of the region-level errors comparing the errors between inputs and reference vs. DL and reference, for AC task.

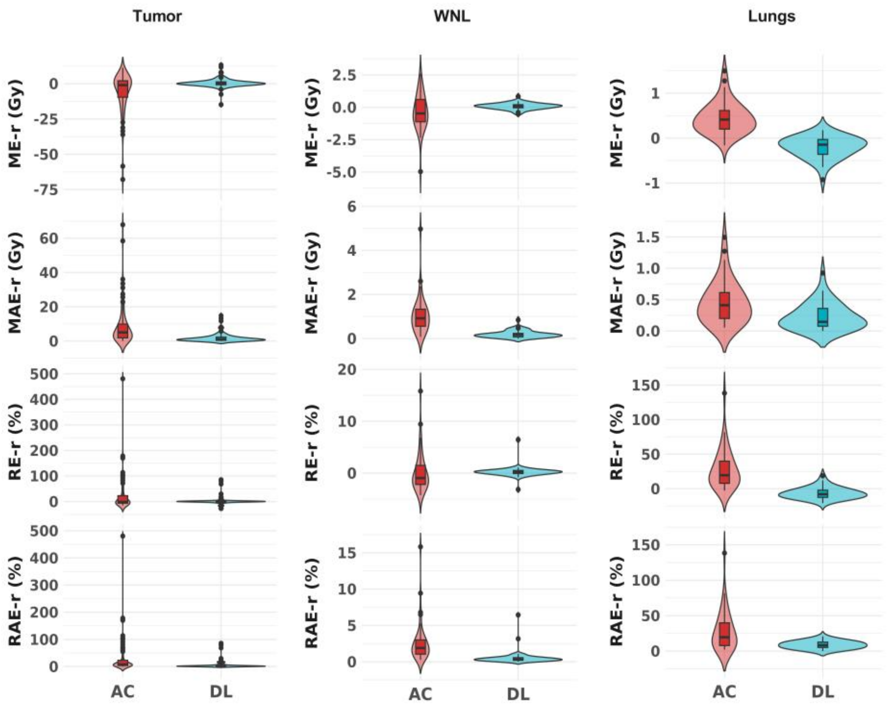

Supplemental-Figure 2. The distribution of the region-level errors comparing the errors between inputs and reference vs. DL and reference, for SC task.

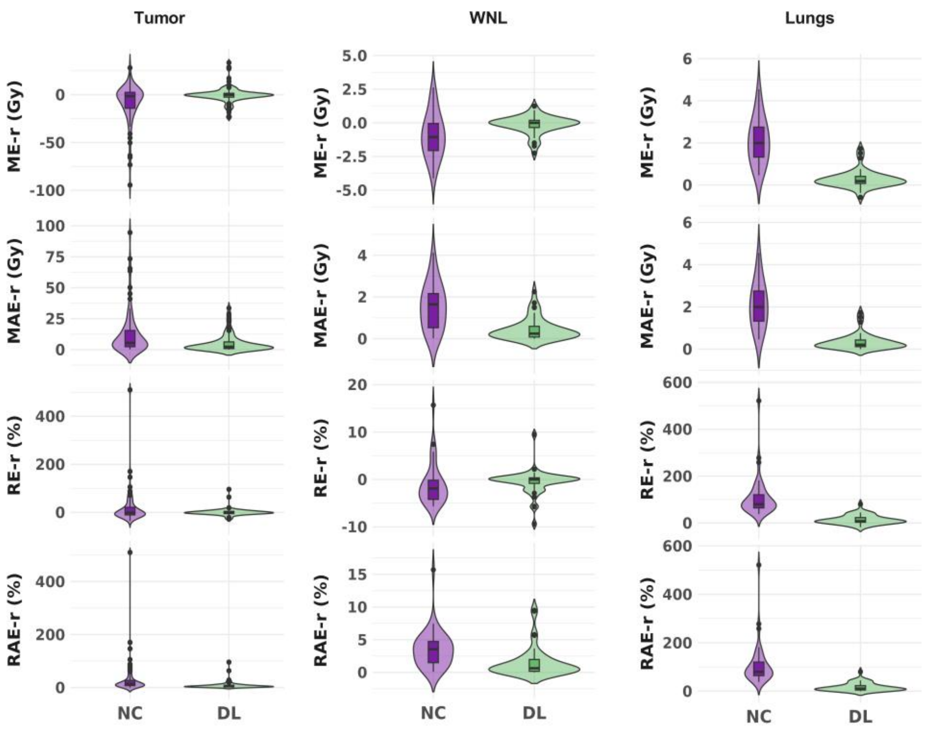

Supplemental-Figure 3. The distribution of the region-level errors comparing the errors between inputs and reference vs. DL and reference, for ASC task.

Supplementary-Table 1. The average± SD of Gamma pass rates comparing the input and their reference of each task. DTA: distance-to-agreement (mm) and DD: dose difference (%), WNL: whole normal liver.

DTA & DD	4.795 mm & 1%			10mm & 5%			15mm & 10%		
	AC	SC	ASC	AC	SC	ASC	AC	SC	ASC
Whole image	99.12 ± 0.57	99.35 ± 0.30	98.96 ± 0.56	99.87 ± 0.08	99.91 ± 0.05	99.84 ± 0.091	99.96 ± 0.03	99.99 ± 0.01	99.96 ± 0.02
Tumor	97.18 ± 1.39	96.83 ± 1.13	96.94 ± 1.27	98.83 ± 1.36	99.05 ± 1.03	98.98 ± 1.11	99.48 ± 0.93	99.91 ± 0.17	99.68 ± 0.49
WNL	97.96 ± 0.72	97.79± 0.68	97.90 ± 0.67	99.21 ± 0.43	99.47 ± 0.27	99.38 ± 0.34	99.69 ± 0.24	99.93 ± 0.06	99.83 ± 0.16
Lungs	52.05 ± 24.02	80.51 ± 12.14	46.46± 21.90	96.62± 3.94	99.22± 1.59	92.61± 7.48	99.73 ± 0.71	99.99 ± 0.01	99.35 ± 1.27

Supplemental Table 2. The mean absorbed dose (MAD) ± standard deviation (SD) for each organ derived from the Reference, Input, and DL-based dose maps. Mann-Whitney U tests were used to compare values across all tasks, with p-values reported for each organ. A p-value of less than 0.05 was interpreted as statistically significant.

Task	Organs	Reference (MAD ± SD)	Input MAA (MAD ± SD)	P-value (input- MAA)	DL (MAD ± SD)	P-value (DL)
AC	Tumour	102.7 ± 100	99.6 ± 100	>0.05	102.7 ± 100	>0.05
	WNL	48.31 ± 24	47.71 ± 24	>0.05	48.03 ± 24	>0.05
	Lungs	3.252 ± 2.2	4.902 ± 3.1	**<0.05**	3.574 ± 2.4	>0.05
SC	Tumour	108.4 ± 110	102.7 ± 100	>0.05	108.9 ± 110	>0.05
	WNL	48.65 ± 25	48.31 ± 24	>0.05	48.74 ± 25	>0.05
	Lungs	2.808 ± 2.1	3.252 ± 2.2	>0.05	2.608 ± 1.9	>0.05
ASC	Tumour	108.4 ± 110	99.6 ± 100	**< 0.05**	108.3 ± 110	>0.05
	WNL	48.65 ± 25	47.71 ± 24	>0.05	48.5 ± 25	>0.05
	Lungs	2.808 ± 2.1	4.902 ± 3.1	>0.05	3.086 ± 2.3	>0.05
